# Integrating network toxicology, machine learning, and single-cell sequencing to reveal the FASN-mediated role of phenolic endocrine disruptors in water in promoting prostate cancer

**DOI:** 10.1371/journal.pone.0350638

**Published:** 2026-06-02

**Authors:** Xinyao Zhu, Qilong Wu, Yuqi Li, Zhiyu Liu, Yang Zeng, Zhiqiang Zeng, Yubo Zhou, Lunhong Zou, Xiaochun Wu, Dan Zhao, Qingfu Deng, Tao Zhou

**Affiliations:** 1 Department of Urology, Santai Hospital Affiliated to North Sichuan Medical College, Mianyang, Sichuan, China; 2 Department of Urology, Affiliated Hospital of Southwest Medical University, Luzhou, Sichuan, China; 3 Department of Nursing, Santai Hospital Affiliated to North Sichuan Medical College, Mianyang, Sichuan, China; University of Agriculture in Krakow, POLAND

## Abstract

**Background:**

Phenolic endocrine-disrupting chemicals (EDCs) like nonylphenol (NP) and octylphenol (OP) are widespread water pollutants. Their estrogen-like properties are suspected contributors to prostate cancer, but their precise molecular mechanisms remain unclear.

**Methods:**

We employed a multidimensional framework to investigate this link. Potential NP/OP targets were predicted using SwissTargetPrediction, SEA, and CTD databases and cross-referenced with prostate cancer-associated genes from GeneCards and OMIM. Differential expression analysis of the GSE46602 dataset (36 tumor vs. 14 benign samples) identified candidate genes, which were refined to core genes using Least Absolute Shrinkage and Selection Operator (LASSO) and Support Vector Machine-Recursive Feature Elimination (SVM-RFE) algorithms. Their diagnostic power was evaluated via an Artificial Neural Network (ANN) model and validated in The Cancer Genome Atlas (TCGA) cohort. Single-cell RNA sequencing data from six prostate cancer samples (GSE137829) were analyzed to reveal cell-type-specific expression patterns. Molecular docking and molecular dynamics (MD) simulations assessed binding stability between pollutants and target proteins.

**Results:**

We identified 143 overlapping genes between NP/OP targets and prostate cancer-associated genes, significantly enriched in lipid metabolism and prostate cancer pathways (adjusted P < 0.05). Dual-algorithm screening identified four core genes (ENPP2, FASN, PTGS2, and CHRM1). Among them, Fatty Acid Synthase (FASN) exhibited the best diagnostic performance in the TCGA validation cohort (AUC = 0.800), outperforming PTGS2 (0.783), ENPP2 (0.621), and CHRM1 (0.605), and was significantly overexpressed in prostate cancer tissues (|log2FC| > 1, adjusted P < 0.05). Single-cell analysis across seven annotated cell types revealed specific FASN overexpression in epithelial cells, with expression progressively upregulated along pseudotime disease trajectories. Gene Set Variation Analysis (GSVA) demonstrated significant activation of oncogenic pathways — including PI3K-AKT-mTOR, androgen response, and early estrogen response — in FASN-high epithelial cells. Molecular docking confirmed favorable binding of NP and OP to FASN (binding affinities of −6.0 and −6.1 kcal/mol, respectively), and MD simulations showed that both complexes reached stable equilibrium with RMSD fluctuations below 0.3 nm. Molecular Mechanics/Generalized Born Surface Area (MM/GBSA) calculations further yielded binding free energies of −19.70 kcal/mol (NP-FASN) and −17.24 kcal/mol (OP-FASN).

**Conclusion:**

This study computationally identifies FASN as a potential molecular hub that may link phenolic EDC exposure to prostate cancer. Our bioinformatic analyses suggest a hypothetical mechanism involving pollutant-driven disruption of lipid metabolic reprogramming *via* FASN, potentially activating a pro-oncogenic network, which warrants future experimental validation.

## Introduction

Prostate cancer has emerged as a major malignancy threatening men’s health globally, with its incidence continuously rising [1]. In addition to age, ethnicity, and genetic factors, accumulating epidemiological evidence points to the significant role of environmental exposures in the disease process [2]. However, the molecular mechanisms by which environmental pollutants may potentially contribute to prostate carcinogenesis remain to be systematically elucidated.

Among surface water pollutants, phenolic endocrine-disrupting chemicals (EDCs) have garnered considerable attention due to their widespread occurrence and endocrine-disrupting activity [3]. As degradation products of industrial surfactants, nonylphenol (NP) and octylphenol (OP) have been ubiquitously detected in water bodies worldwide [4]. Of particular concern, the average concentrations of NP and OP in surface waters of some regions in China have exceeded the European Union’s environmental safety limits, indicating non-negligible exposure risks for the population through drinking water and dermal contact [5].

Although existing studies suggest that phenolic EDCs may contribute to the development of hormone-dependent cancers *via* estrogen-mimicking effects, significant knowledge gaps remain regarding the specific mechanisms of NP/OP in prostate cancer [6]. Notably, several prior studies have provided valuable mechanistic insights: Forte et al. demonstrated that NP acts on prostate adenocarcinoma cells through estrogen receptor (ER) molecular pathways, modulating proliferation and apoptosis [7]; Di Lorenzo et al. further elucidated that NP affects prostate cancer cell cycle regulation via ERα/ERβ-mediated mechanisms [8]; early work by Hess-Wilson et al. revealed that alkylphenolic xenoestrogens can influence androgen receptor signaling in prostate cells [9]. These studies collectively establish that NP/OP exerts biological effects on prostate cells primarily through classical hormone receptor pathways. However, most research has focused on bisphenol A (BPA), while systematic investigations into NP/OP are relatively scarce. More importantly, current understanding is largely confined to the classical pathway of EDCs “directly binding to hormone receptors.” Whether NP/OP can also induce metabolic reprogramming by directly interacting with key metabolic enzymes (e.g., lipid synthases), representing a hormone receptor-independent mechanism, and subsequently co-activate oncogenic signaling networks remains poorly explored. This lack of insight into the ‘metabolism-hormone’ interplay limits a comprehensive understanding of the potential cancer-related effects of environmental pollutants.

Traditional toxicology, constrained by its single-target approach, struggles to address the multi-pathway characteristics of pollutants and the cellular heterogeneity of the tumor microenvironment, thereby impeding research into the etiology of environmental exposure. Emerging technology combinations offer significant advantages: the integration of network toxicology and machine learning can efficiently construct pollutant-target-disease association networks and improve prediction efficiency; single-cell transcriptome analysis captures gene expression differences across cell subpopulations and reveals heterogeneous responses; molecular simulation validation corroborates interactions at the atomic level. Together, these approaches enable in-depth mechanistic breakthroughs. These synergistic technologies form a cohesive “prediction–analysis–validation” pipeline, overcoming traditional bottlenecks and providing more precise and comprehensive technical support for environmental toxicology.

To address these scientific questions, this study established an interdisciplinary technical framework integrating network toxicology prediction, machine learning screening, single-cell transcriptome analysis, molecular simulation validation to systematically investigate the potential molecular mechanisms by which NP/OP may be associated with prostate cancer progression. Through this integrated strategy, we aim to: (1) identify key molecular targets of NP/OP in regulating prostate cancer; (2) elucidate the regulatory axis of “pollutant exposure-metabolic reprogramming-oncogenic signaling activation”; and (3) provide a theoretical basis for the precise prevention and treatment of environment-associated prostate cancer.

## Method

### Pollutant target prediction and disease gene integration

The chemical structures and SMILES identifiers of NP and OP were retrieved from the PubChem database (https://pubchem.ncbi.nlm.nih.gov) and imported into the SwissTargetPrediction (http://www.swisstargetprediction.ch/), SEA (https://sea.bkslab.org/), and CTD platforms (https://ctdbase.org/) for target prediction. The results were merged and deduplicated to construct a pollutant target dataset. Simultaneously, prostate cancer-related genes were extracted from the GeneCards (screening score > 1) (https://www.genecards.org/) and OMIM databases (https://www.omim.org/). After integration and deduplication, a disease target set was obtained. The intersection of pollutant targets and disease targets yielded the NP/OP-Prostate Cancer common target genes.

### Functional enrichment analysis

KEGG pathway enrichment analysis of the intersection genes was performed using the R package clusterProfiler (p < 0.05 and p.adjust < 0.05), and results were visualized *via* ggplot2. GO functional enrichment analysis, covering biological process, cellular component, and molecular function categories, was conducted with a significance threshold of p < 0.05 and p.adjust < 0.05. A gene-GO term chord diagram was generated using the circlize package.

### Data acquisition and study strategy

To identify robust prostate cancer-associated gene expression signatures, we searched the Gene Expression Omnibus (GEO) database (https://www.ncbi.nlm.nih.gov/geo/) using the keywords ‘prostate cancer’ AND ‘benign prostate’. The search results were filtered for ‘Homo sapiens’. Among the eligible datasets, the GSE46602 dataset was selected for downstream analysis. This dataset comprises a total of 50 clinical specimens, explicitly including 36 prostate adenocarcinoma tissues and 14 matched benign prostate tissue samples. To ensure the reliability of the results, we employed a comprehensive screening strategy: Machine Learning algorithms were applied to identify robust diagnostic features, which were subsequently validated using an independent single-cell transcriptomic dataset (GSE137829) to confirm their cell-type specific expression patterns.

### Differentially expressed gene screening

Data pre-processing was performed as follows: First, probe IDs were mapped to standard gene symbols based on the platform annotation files. For genes represented by multiple probes, the arithmetic mean of the expression values was calculated to represent the final expression level. To ensure data comparability and minimize systematic bias, the expression matrix was normalized using the Quantile Normalization method implemented in the limma package. Furthermore, to enhance statistical power and reduce the false discovery rate, we performed low-expression filtering by excluding genes with average expression levels ranking in the lowest 30%. Subsequently, the linear models for microarray data (limma) package in R was utilized to identify differentially expressed genes (DEGs) between prostate cancer and benign tissues. The Empirical Bayes method was applied to moderate t-statistics, and P-values were adjusted using the Benjamini-Hochberg (BH) procedure. Genes meeting the criteria of |log2FC| > 1 and adjusted P-value < 0.05 were considered statistically significant DEGs.

### Machine learning-based core gene screening

To screen for the most valuable diagnostic features and minimize the risk of overfitting, we employed an integrated strategy combining two machine learning algorithms: Least Absolute Shrinkage and Selection Operator (LASSO) regression and Support Vector Machine-Recursive Feature Elimination (SVM-RFE).

LASSO Regression Analysis: The LASSO logistic regression model was constructed using the ‘glmnet’ R package (family = ‘binomial’). To determine the optimal penalty parameter λ, we performed 10-fold cross-validation. The λmin value (minimizing binomial deviance) was selected to identify genes with non-zero coefficients. L1 regularization was applied to effectively shrink irrelevant coefficients, thereby preventing overfitting.

SVM-RFE Feature Selection: SVM-RFE analysis was conducted using the ‘e1071’ package. Gene expression data first underwent Z-score standardization. We utilized an SVM with a Linear Kernel (initial cost = 10) as the classifier. Feature importance was ranked *via* Recursive Feature Elimination with 10-fold cross-validation. Within each fold, Grid Search was applied to optimize hyperparameters (Gamma and Cost). The feature subset yielding the lowest Cross-Validation Error and highest Accuracy was selected.

ANN Construction & Evaluation: The intersection of genes from LASSO and SVM-RFE was defined as the core gene set. For the Artificial Neural Network (ANN) construction using the ‘neuralnet’ package, expression data was first binarized: expression values greater than the median were encoded as 1 (High), and others as 0 (Low). The network architecture consisted of one hidden layer with 5 neurons and an output layer with two nodes (Tumor vs. Normal). The classification performance was evaluated using the Confusion Matrix to calculate Accuracy, and the discriminative ability was assessed *via* the Area Under the Receiver Operating Characteristic Curve (AUC).

### Single-cell transcriptome analysis

The GSE137829 dataset was processed using the standard Seurat workflow: normalization, selection of highly variable genes, PCA dimensionality reduction, and t-SNE visualization. Batch effect correction was performed using the harmony package. Cell types were annotated into subpopulations—epithelial, endothelial, fibroblast, myofibroblast, myeloid, B cell, and T cell—based on classical cell marker genes:T cells: CD3D, CD3E, CD2, CD3G; Myeloid cells: CD68, CD14, AIF1, CSF1R; B cells: CD79A, CD79B, MS4A1, CD19; Fibroblasts: DCN, TNFAIP6, APOD, FBLN1; Endothelial cells: ENG, VWF, CLDN5, CDH5; Epithelial cells: CDH1, EPCAM, KRT8, KRT5; Myofibroblasts: MYH11, GJA4, RGS5, MT1A [10].

Differentially expressed genes for each cell cluster were identified using the FindAllMarkers function. Pseudotime analysis was conducted with the monocle package to trace cell state transition trajectories and decipher the dynamic expression pattern of FASN during disease progression. Epithelial cells were divided into high- and low-expression groups based on the median FASN expression level, and pathway activation differences between the two groups were compared using Gene Set Variation Analysis (GSVA) analysis.

### Molecular docking

The 3D structure of human full-length Fatty Acid Synthase (FASN) was retrieved from the RCSB Protein Data Bank (https://www.rcsb.org/, PDB ID: 8VF7), which was resolved by electron microscopy. And the 3D structures of the ligands, NP and OP, were obtained from PubChem (https://pubchem.ncbi.nlm.nih.gov). The protein structure was prepared using AutoDockTools 1.5.7 by removing water molecules, adding polar hydrogen atoms, and computing Gasteiger charges. Molecular docking simulations were performed using AutoDock Vina.

A two-step docking strategy was employed to identify the binding sites. First, blind docking was performed over the entire surface of the full-length FASN protein, with the grid box dimensions set to encompass the whole protein to allow unbiased sampling of all potential binding regions. The top-ranked binding poses for NP and OP were analyzed, and the most energetically favorable binding regions were identified. Subsequently, refined docking was conducted by centering the grid boxes on the binding regions identified from the blind docking results. For the NP-FASN complex, the grid center was set at x = 181.056, y = 220.471, z = 150.005, with grid dimensions of 35 × 35 × 35 Å. For the OP-FASN complex, the grid center was configured at x = 221.323, y = 184.874, z = 119.166, with dimensions of 31 × 22 × 29 Å. Binding modes with an affinity score lower than −5 kcal/mol were considered to indicate stable molecular interactions. The best binding poses were visualized using PyMOL.

### Dynamics simulations

Molecular dynamics (MD) simulations were performed using the GROMACS 2023 package with GPU acceleration. The Amber99sb force field was selected to describe the protein-ligand interactions due to its high accuracy in reproducing protein conformational properties, coupled with the TIP3P water model for solvent description.

System Setup and Minimization: The protein-ligand complex was immersed in a cubic solvation box with a minimum distance of 1.0 nm between the solute and the box edges to prevent periodic boundary artifacts. The system was neutralized and adjusted to a physiological salt concentration of 0.15 M NaCl. A two-stage energy minimization strategy was employed: first using the Steepest Descent algorithm to remove steric clashes, followed by the BFGS algorithm (convergence threshold < 1000 kJ/mol/nm) to fine-tune the structure.

Equilibration and Production: The system underwent NVT equilibration (100 ps) using the V-rescale thermostat to maintain the temperature at 300 K, followed by NPT equilibration (100 ps) using the Parrinello-Rahman barostat to maintain pressure at 1 bar. Position restraints (1000 kJ/mol/nm²) were applied to the ligand and protein heavy atoms during equilibration to stabilize the binding mode. Finally, a 50 ns production run was conducted without restraints. The time step was set to 2 fs, with bond lengths constrained by the LINCS algorithm. Long-range electrostatic interactions were calculated using the Particle Mesh Ewald (PME) method (cutoff 1.2 nm).

Trajectory Analysis: The trajectory was corrected for periodic boundary conditions using the trjconv tool. Structural stability was evaluated via Root Mean Square Deviation (RMSD, reflecting overall structural stability and equilibration of the complex), Root Mean Square Fluctuation (RMSF, indicating local residue flexibility, with higher values representing more mobile regions), Radius of Gyration (Rg, measuring protein compactness and folding integrity), Solvent Accessible Surface Area (SASA, assessing conformational changes in protein surface exposure), and Hydrogen Bond analysis (evaluating the persistence and strength of protein-ligand interactions) using the built-in GROMACS tools. The Free Energy Landscape (FEL) was constructed based on Rg and RMSD variables to identify the most energetically favorable conformational states and assess conformational stability.

### Binding free energy calculation

To quantitatively assess the interaction strength between the ligands (NP/OP) and the FASN protein, the binding free energies were calculated using the Molecular Mechanics/Generalized Born Surface Area (MM/GBSA) method implemented in the gmx_MMPBSA v1.6.3 tool. The calculations were performed based on the stable equilibrium trajectory extracted from the MD simulations. The binding free energy (ΔGbind) was estimated using the following equation:ΔGbind = ΔGcomplex −(ΔGreceptor +ΔGligand).where ΔGcomplex, ΔGreceptor, and ΔGligand represent the free energies of the complex, receptor, and ligand, respectively. The energy terms include van der Waals interactions, electrostatic interactions, polar solvation energy (calculated *via* the Generalized Born model), and non-polar solvation energy.

### Ethical statement

The data used in this study were obtained from publicly available databases, including GeneCards and the Gene Expression Omnibus (GEO). Since these databases consist of anonymized data that is freely accessible to the public, no ethical approval or informed consent was required for this study. The data were used solely for research purposes and were in compliance with the relevant ethical guidelines for the use of publicly available data.

## Result

### Construction of the NP/OP-prostate cancer target gene profile

**Fig 1:**
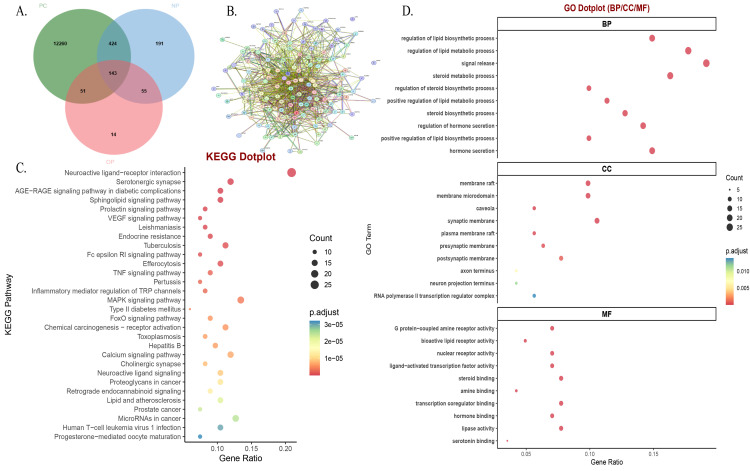
**A.** Venn diagram illustrating the intersection between target genes of Nonylphenol (NP)/Octylphenol (OP) and prostate cancer-related genes. **B.** Protein-protein interaction (PPI) network of the 143 common target genes, constructed using the STRING database. **C.** KEGG pathway enrichment analysis of the common target genes, showing significant enrichment in pathways such as Endocrine resistance, Prostate cancer, and lipid metabolism-related pathways. **D.** Gene Ontology (GO) functional enrichment analysis of the common target genes (Biological Process, Cellular Component, Molecular Function).

We obtained the chemical formulas, molecular weights, and SMILES identifiers of NP and OP from the PubChem database (Table 1). Potential target genes for NP and OP were predicted using SwissTargetPrediction, SEA, and CTD, respectively. Following intersection with the prostate cancer disease gene set (GeneCards & OMIM), a total of 143 common target genes were identified (Fig 1A). To analyze interactions at the protein level, these target genes were mapped to their corresponding proteins. The PPI network constructed using the STRING database revealed that these proteins form a tightly interconnected molecular interaction module (Fig 1B), suggesting functional synergy.

**Table 1:** Name, SMILES Structure, CAS Number, Molecular Formula and Molecular Weight of Phenolic Substances.

Name	SMILES structures	CAS	Molecular Formula	Molecular Weight
Nonylphenol	CCCCCCCCCC1 = CC = CC = C1O	25154-52-3	C_15_H_24_O	220.35g/mol
Octylphenol	CCCCCCCCC1 = CC = CC = C1O	949-13-3	C_14_H_22_O	206.32g/mol

This table summarizes the name, Simplified Molecular Input Line Entry System (SMILES）structure, CAS number, molecular formula and molecular weight of two phenolic substances (Nonylphenol and Octylphenol).

### Functional enrichment points to lipid metabolic dysregulation

KEGG enrichment analysis showed that the intersection genes were significantly enriched in pathways such as Endocrine resistance, TNF signaling pathway, MAPK signaling pathway, Chemical carcinogenesis – receptor activation, and Prostate cancer (Fig 1C). GO analysis further revealed that the primary biological processes involved lipid biosynthesis and the regulation of hormone secretion. Cellular components were enriched in membrane rafts and vesicle lumens, while molecular functions were concentrated in G protein-coupled receptor activity and hormone binding (Fig 1D). These results collectively suggest a potential central role of lipid metabolism disruption in the predicted association between NP/OP and prostate cancer.

### Machine learning identifies FASN as a key core gene

**Fig 2:**
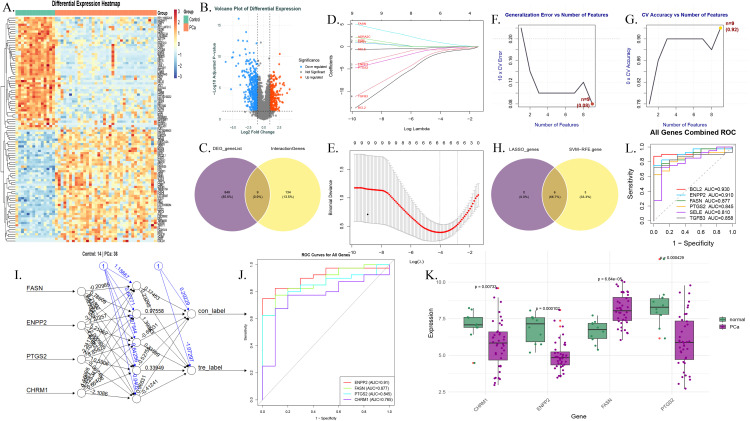
**A-B.** Heatmap (A) and volcano plot (B) of differentially expressed genes from the GSE46602 dataset. D-E. LASSO regression analysis results: coefficient profile plot (D) and 10-fold cross-validation for selecting the optimal lambda value **(E)**, identifying 5 candidate genes with non-zero coefficients. F-G. SVM-RFE analysis results: Relationship between generalization error and number of features (F) and relationship between cross-validation accuracy and number of features **(G)**. Based on the combined performance of generalization error and accuracy, 4 candidate genes were screened. **H.** Venn diagram showing the intersection of genes identified by LASSO regression and SVM-RFE, resulting in 4 core genes (ENPP2, FASN, PTGS2, CHRM1). I-J. Artificial Neural Network (ANN) model performance: Model architecture with one hidden layer (5 neurons) (I) and ROC curve validation **(J)**, demonstrating the model’s discriminative ability for prostate cancer. **K.** Expression level of FASN in prostate cancer tissues versus benign tissues, showing its significant overexpression. **L.** Diagnostic performance of FASN in the TCGA cohort, indicating its superior diagnostic capability among the core genes.

Differential analysis of the GSE46602 dataset yielded significantly differentially expressed genes, with a heatmap and volcano plot clearly illustrating their expression distribution patterns (Fig 2A-B). Intersecting these differentially expressed genes with the common target genes screened 7 key candidate genes. LASSO regression selected 5 genes (Fig 2D-E), while SVM-RFE identified 4 genes (Fig 2F-G). The intersection of these two gene sets yielded four core genes: ENPP2, FASN, PTGS2, and CHRM1 (Fig 2H). The structure of the ANN model and the ROC curve verification demonstrated that this four-gene combination has a strong discriminative capacity for prostate cancer (Fig 2I-J). Among them, FASN exhibited the best diagnostic performance in the TCGA cohort (Fig 2L) and was significantly overexpressed in prostate cancer tissues (Fig 2K), thus being identified as the core target gene.

### Single-cell resolution reveals cellular heterogeneity of FASN expression

**Fig 3:**
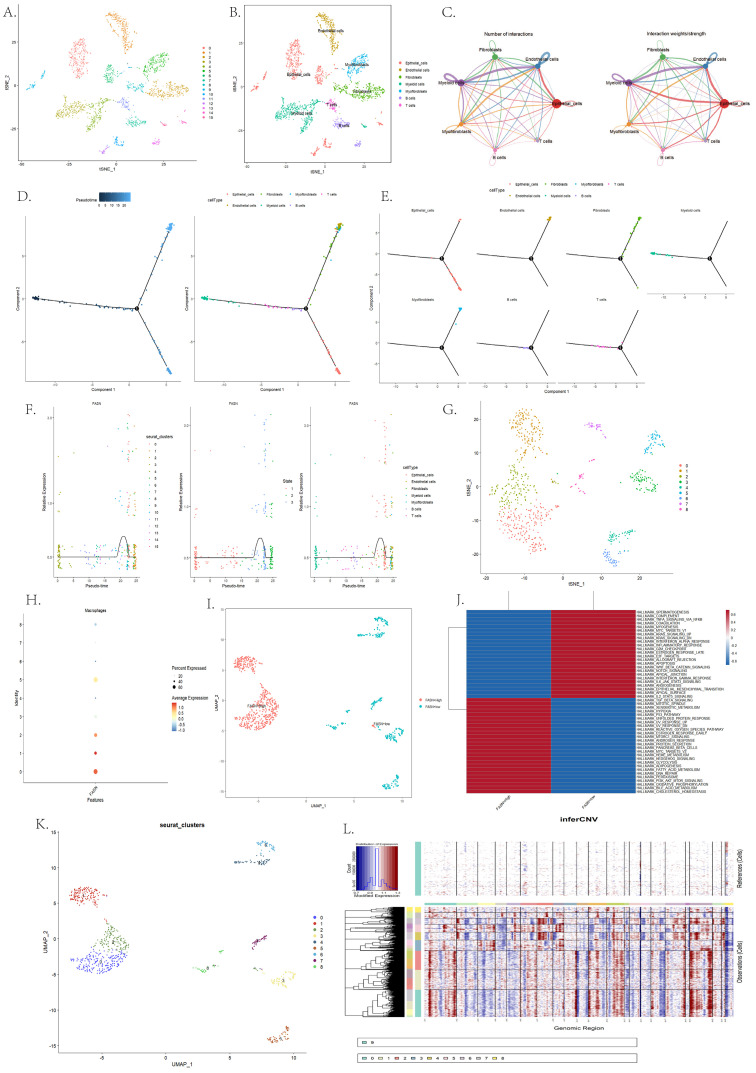
**A-B.** t-SNE visualization of the GSE137829 dataset after quality control and batch effect correction (using the Harmony package) (A), and annotation of the seven major cell types: Epithelial cells, Endothelial cells, Fibroblasts, Myofibroblasts, Myeloid cells, B cells, and T cells (B). **C.** Cell communication analysis, revealing epithelial cells as the central hub within the signaling network. D-E. Pseudotime analysis results: Three distinct disease progression states (D) and the dynamic changes in the proportions of cell types (Epithelial cells, Endothelial cells, Fibroblasts) within the advanced state **(E). F.** Dynamic expression trajectory of FASN along the pseudotime axis, showing its specific enrichment and gradual upregulation in epithelial cells. G-J. Comparative GSVA pathway enrichment analysis between FASN-high and FASN-low epithelial cell groups, highlighting the activation of oncogenic pathways including the PI3K-AKT-mTOR signaling pathway, Androgen Response, and Early Estrogen Response **(G-J)**. K-L. InferCNV heatmap depicting inferred chromosomal copy number variations (CNVs) in epithelial cells, with T cells as the reference (upper panel). The lower panel shows CNV profiles of epithelial subpopulations (clusters 0–8). Red and blue indicate chromosomal gains and losses, respectively. FASN-high clusters exhibited elevated genomic instability compared to the reference population.

After quality control and batch effect correction of the GSE137829 single-cell data, seven major cell types were annotated, encompassing epithelial, endothelial, fibroblast, myofibroblast, myeloid, B cell, and T cells (Fig 3A-B). Cell communication analysis indicated that epithelial cells occupy a central hub within the signaling network (Fig 3C). Pseudotime analysis identified three distinct disease progression states, with the proportions of epithelial, endothelial, and fibroblast cells significantly increasing in the late state (Fig 3D-E). FASN expression was progressively upregulated along the pseudotime trajectory and showed specific enrichment in epithelial cells (Fig 3F). Dividing epithelial cells into high and low groups based on the median FASN expression level, GSVA analysis revealed significant activation of multiple cancer-promoting pathways in the FASN-High group, including TGF-β signaling, p53 pathway, Reactive oxygen species, Early estrogen response, Androgen response, Glycolysis, and PI3K-AKT-mTOR signaling (Fig 3G-J), suggesting a strong association between high FASN expression and a malignant phenotype. Furthermore, inferCNV analysis was performed on the sub-clustered epithelial cells using T cells as the reference. The results revealed that FASN-high epithelial clusters (clusters 0, 1, and 2) exhibited a greater propensity for chromosomal copy number alterations compared to other subpopulations (Fig 3K-L), further supporting the association between elevated FASN expression and increased tumor cell malignancy.

### Molecular simulations suggest direct NP/OP-FASN binding potential

**Fig 4:**
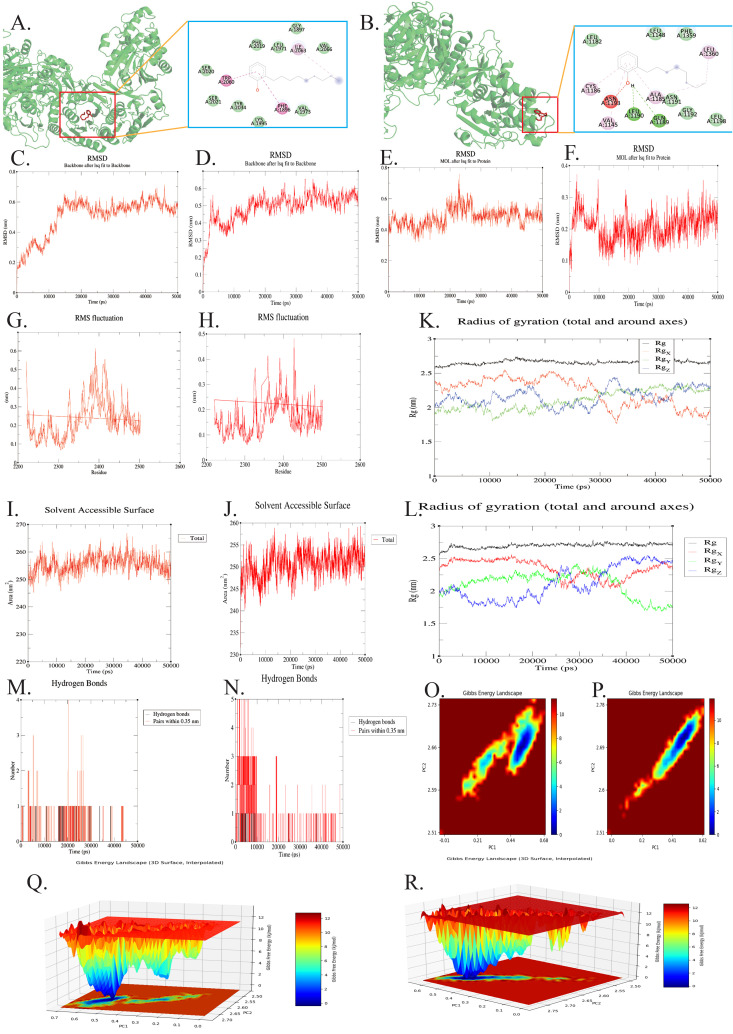
**A-B.** Molecular docking models showing the binding conformations of NP (A) and OP (B) with FASN (binding affinity: NP, −6.0 kcal/mol; OP, −6.1 kcal/mol).C-D, Backbone root mean square deviation (RMSD) plots of the FASN–NP (C) and FASN–OP (D) complexes during the molecular dynamics simulations. E-F, Ligand RMSD plots of NP (E) and OP (F) after fitting to the protein. G-H, Root mean square fluctuation (RMSF) profiles of FASN residues in the FASN–NP (G) and FASN–OP (H) complexes. I-J, Solvent accessible surface area (SASA) plots of the FASN–NP (I) and FASN–OP (J) complexes. K-L, Radius of gyration (Rg) plots of the FASN–NP (K) and FASN–OP (L) complexes. M-N, Hydrogen bond analysis of the FASN–NP (M) and FASN–OP (N) complexes during the simulation. O-P, Two-dimensional free energy landscapes (FELs) of the FASN–NP (O) and FASN–OP (P) complexes. Q-R, Three-dimensional free energy landscapes (FELs) of the FASN–NP (Q) and FASN–OP (R) complexes.

Molecular docking showed binding energies of −6 kcal/mol and −6.1 kcal/mol for FASN with NP and OP, respectively, indicating favorable binding affinities. To further elucidate the binding mechanisms, we visualized the intermolecular interactions using Discovery Studio. The 2D interaction analysis revealed distinct binding modes for the two ligands. For the OP-FASN complex, stability is primarily maintained through hydrogen bonds formed with residues GLN1189 and LEU1190, alongside Pi-Alkyl/Alkyl hydrophobic interactions involving ALA1185, CYS1186, and LEU1360. PHE1359 provides supportive Van der Waals contacts. These residues are located within the non-catalytic structural region between the DH domain (residues 838–1108) and the ER domain (residues 1635–1863), encompassing the pseudo-methyltransferase (ψME) domain and its flanking linker region. This region lacks intrinsic enzymatic activity but serves as a structural scaffold essential for maintaining the dimeric architecture and inter-domain spatial organization of FASN. In contrast, the interaction between NP and FASN is dominated by hydrophobic forces, specifically Pi-Pi T-shaped interactions with residues TRP2060 and PHE1896, and Alkyl interactions with ILE2063. SER2020 and TYR2034 provide supportive Van der Waals contacts. These residues are located within the β-ketoacyl reductase (KR) domain (residues 1864–2118), which catalyzes the NADPH-dependent reduction of β-ketoacyl intermediates during the fatty acid elongation cycle (Fig 4A-B). Molecular dynamics simulations demonstrated that both FASN–NP and FASN–OP complexes reached equilibrium after approximately 15–20 ns. The backbone RMSD stabilized at 0.56 ± 0.05 nm for the FASN–NP complex and 0.54 ± 0.05 nm for the FASN–OP complex, indicating overall structural stability (Fig 4C-D). The ligand RMSD for NP fluctuated around 0.48 ± 0.06 nm, whereas OP showed a lower fluctuation of 0.22 ± 0.05 nm, suggesting a more stable binding conformation for OP within the FASN pocket (Fig 4E-F). RMSF analysis showed limited residue-level flexibility, with average fluctuations of 0.22 ± 0.08 nm (NP) and 0.20 ± 0.07 nm (OP) (Fig 4G-H). Although several loop regions exhibited higher mobility, no global conformational instability was observed (Fig 4I-J). The radius of gyration (Rg) remained stable at 2.66 ± 0.04 nm (NP) and 2.70 ± 0.03 nm (OP), while SASA values fluctuated within narrow ranges (255.3 ± 4.2 nm² for NP and 251.5 ± 3.5 nm² for OP), indicating that both complexes maintained compact conformations (Fig 4K-L). Hydrogen bond analysis revealed relatively stable interactions, with averages of 0.8 ± 0.6 (NP) and 1.2 ± 0.9 (OP), suggesting that hydrophobic interactions dominate in the NP system, whereas both hydrogen bonding and hydrophobic interactions contribute to OP binding (Fig 4M-N). Furthermore, free energy landscape (FEL) analysis revealed that both FASN–NP and FASN-OP complexes exhibited a single dominant low-energy basin, indicating that the systems converged to a thermodynamically stable conformational state (Fig 4O-R). Quantitative analysis using MM/GBSA further validated the binding strength. NP demonstrated a higher binding affinity with a ΔGbind of −19.70 kcal/mol, whereas OP showed a slightly lower affinity of −17.24 kcal/mol. Collectively, these results indicate that both NP and OP can stably bind to FASN under dynamic conditions, with OP exhibiting greater conformational stability and NP showing slightly stronger binding affinity.

## Discussion

Integrated multi-omics and computational biology approaches in this study identified FASN as a potential key molecular node that may be associated with NP/OP exposure and prostate cancer progression. This hypothesis provides a novel theoretical framework for understanding the pathogenesis of environment-associated prostate cancer.

The identification of FASN was facilitated by a cross-validated multi-algorithm strategy. Network toxicology narrowed the target scope to 143 overlapping genes. Functional enrichment analysis pinpointed lipid metabolism dysregulation, aligning with the theory of cancer metabolic reprogramming. Machine learning further refined core targets from differentially expressed genes, wherein FASN demonstrated diagnostic superiority in the model, consistent with its previously reported pro-tumorigenic functions [11,12]. Single-cell analysis revealed the heterogeneous expression of FASN at cellular resolution—its specific upregulation in epithelial cells and association with advanced disease states suggest its potential involvement in disease progression, although a causal role remains to be experimentally validated.

The epithelial cell-specific overexpression of FASN precisely corresponds to the pathophysiological characteristics of prostate cancer, which originates from prostate epithelial cells and relies heavily on metabolic reprogramming—particularly heightened *de novo* lipogenesis—to meet the energy and biomaterial demands of rapid proliferation [12,13]. Previous studies have established that FASN overexpression-driven fatty acid synthesis is a characteristic feature and therapeutic target in prostate cancer [12,14]. Critically, FASN-mediated lipid metabolism forms a tightly coordinated regulatory axis with androgen signaling, which is decisive in prostate cancer progression. As an androgen-dependent malignancy, sustained Androgen Receptor (AR) activation is a core oncogenic mechanism [15,16]. FASN-mediated lipogenesis enhances AR nuclear localization and transcriptional activity, potentially regulating resistant variants like AR-V7 [17,18]. Conversely, androgens cooperatively activate SREBF1 *via* AR and mTOR to drive FASN transcription, establishing a positive feedback loop: Androgen–AR–SREBF1–FASN–AR activation [19,20]. Our GSVA analysis showing significant androgen response pathway activation in FASN-high epithelial cells provides single-cell level evidence supporting this regulatory axis, consistent with mechanisms whereby Darolutamide suppresses FASN by downregulating SREBF1 [21]. Notably, NP/OP, as estrogen-mimicking EDCs, might disrupt this axis through dual effects: directly stabilizing FASN enzymatic activity to reinforce lipid metabolic reprogramming, and simultaneously disrupting androgen signaling balance *via* ER–AR crosstalk [7]. This multi-tiered regulatory chain—“Pollutant–FASN–Lipid Metabolism–Androgen Signaling”—may explain how environmental pollutants precisely target susceptible prostate epithelial cells by disrupting the homeostatic balance between metabolism and hormonal signals.

The innovation of this study lies in integrating “metabolic reprogramming” into the theoretical framework of EDC-mediated endocrine disruption. Traditional research focuses on direct EDC–hormone receptor interactions, whereas this study proposes that pollutant–metabolic enzyme interaction might bridge environmental exposure and tumor progression. Molecular docking predicts that NP and OP may bind to distinct functional regions of FASN with favorable affinity, and these interactions were further supported by molecular dynamics simulations and MM/GBSA analysis, which confirmed stable binding under dynamic conditions. NP preferentially binds within the KR domain, which catalyzes the NADPH-dependent reduction step in fatty acid elongation, suggesting that NP may potentially interfere with this catalytic function. OP, in contrast, binds within the non-catalytic ψME structural region situated between the DH and ER catalytic domains. This region is critical for maintaining the overall dimeric architecture and the spatial coordination among catalytic domains required for substrate shuttling by the ACP domain. OP binding at this site may therefore affect FASN structural integrity and conformational dynamics rather than directly inhibiting a specific catalytic step. These distinct binding modes suggest that NP and OP may disrupt FASN function through complementary mechanisms. However, the functional consequences of both binding events require validation through enzymatic activity assays and mutagenesis studies.

Despite the multi-dimensional clues provided by the computational strategy, this study has notable limitations. The primary issue is the lack of direct experimental evidence: neither validation of the regulatory effect of NP/OP exposure on FASN expression in cell or animal models, nor population data supporting the association between exposure levels, FASN expression, and disease risk. Secondly, the static binding mode from molecular simulations cannot reflect the dynamic intracellular environment; the actual binding affinity and functional consequences require confirmation through biochemical assays. Future work should focus on: 1. Verifying the dose-response relationship of NP/OP exposure on FASN expression and activity in prostate cancer cell lines; 2. Assessing the regulatory role of FASN in NP/OP-induced pro-tumorigenic effects using knockdown/overexpression *via* gene editing; 3. Conducting cohort studies to elucidate the association between population NP/OP exposure levels, FASN expression in prostate tissue, and cancer risk. Only through mutual corroboration of experimental and computational findings can the pro-carcinogenic mechanisms of environmental EDCs be fully elucidated, providing a reliable scientific basis for water pollution control and precision prevention.

In summary, this study preliminarily constructs a hypothetical model of “NP/OP–FASN–Prostate Cancer” through multi-technique integration. However, all conclusions are based on bioinformatic inferences; their causal relationships must be verified through *in vitro* functional experiments, *in vivo* exposure models, and epidemiological studies.

## Conclusion

Integrating multidisciplinary technologies—including network toxicology, machine learning, single-cell transcriptome sequencing, and molecular simulation—this study investigated the molecular mechanisms by which phenolic endocrine-disrupting chemicals (NP/OP) may be associated with the development and progression of prostate cancer. The research identified 143 common target genes shared between NP/OP and prostate cancer, which were significantly enriched in lipid metabolism and cancer-related pathways. Through dual-algorithm screening and diagnostic efficacy evaluation, Fatty Acid Synthase (FASN) was identified as a potential key hub gene. FASN exhibited specific high expression in epithelial cells and was closely associated with disease progression trajectories. Molecular docking and molecular dynamics simulations suggest that both NP and OP may potentially bind stably to FASN. Single-cell analysis further revealed that high FASN expression coincides with synergistic activation of multiple oncogenic pathways, including PI3K-AKT-mTOR and androgen/estrogen response. These findings propose a regulatory axis of “NP/OP exposure — FASN-mediated lipid metabolic reprogramming — activation of oncogenic signaling,” offering a novel perspective for understanding the environmental and metabolic etiology of prostate cancer. However, all conclusions in this study are based on bioinformatic inferences, and their causal relationships still require further validation through in vitro cellular experiments, in vivo animal models, and epidemiological investigations. This research lays a theoretical foundation for the control of aquatic pollutants and the development of metabolic-targeted prevention and treatment strategies for prostate cancer.

## Supporting information

S1 Table Target genes of nonylphenol (NP) and octylphenol (OP). (XLSX)

S2 Table Target genes of prostate cancer. (XLSX)
